# Interferon-γ regulates growth and controls Fcγ receptor expression and activation in human intestinal mast cells

**DOI:** 10.1186/1471-2172-15-27

**Published:** 2014-07-05

**Authors:** Gernot Sellge, Miriam Barkowsky, Sigrid Kramer, Thomas Gebhardt, Leif E Sander, Axel Lorentz, Stephan C Bischoff

**Affiliations:** 1 Department of Internal Medicine III, University Hospital Aachen, RWTH University, Aachen, Germany; 2 Department of Obstetrics and Gynecology, Cologne Municipal Health Care, Cologne, Germany; 3 Department of Nutritional Medicine, University of Hohenheim, Stuttgart, Germany; 4 Department of Microbiology and Immunology, The University of Melbourne, Melbourne, Australia; 5 Departmant of Internal Medicine/Infectious Diseases and Respiratory Medicine, Charité - Universitätsmedizin Berlin, Berlin, Germany

**Keywords:** Human, Mast cells, Intestine, Interferon-γ, Fcγ receptors

## Abstract

**Background:**

Development and function of tissue resident mast cells (MCs) is tightly controlled by various cytokines, most of which belong to the typical T helper (Th) 2-type cytokines such as IL-3 and IL-4. The effects of the Th1-type cytokine IFN-γ on human MCs is less clear.

**Results:**

Here, we analyzed the effects of IFN-γ on tissue-derived, mature human MCs. We found that INF-γ decreases proliferation, without affecting apoptosis in human intestinal MCs cultured in the presence of optimal concentrations of stem cell factor (SCF) or SCF and IL-4. However, in the absence of growth factors or at suboptimal concentrations of SCF, INF-γ promotes survival through inhibition of MC apoptosis. Interestingly, we found that INF-γ has no effect on FcϵRI expression and FcϵRI-mediated release of histamine and leukotriene (LT)C_4,_ while it has profound effects on FcγR expression and activation. We show that intestinal MCs express FcγRI, FcγRIIa, and FcγRIIc, whereas FcγRIIb expression was found in only 40% of the isolates and FcγRIII was never detectable. INF-γ strongly increases FcγRI and decreases FcγRIIa expression. INF-γ-naïve MCs produce LTC_4_ but fail to degranulate upon crosslinking of surface-bound monomeric IgG. In contrast, INF-γ-treated MCs rapidly release granule-stored histamine in addition to de novo-synthesized LTC_4_.

**Conclusion:**

In summary, we identify INF-γ as an important regulator of tissue-resident human MCs. IFN-γ displays a dual function by blocking extensive MC proliferation, while decreasing apoptosis in starving MCs and enhancing FcγRI expression and activation. These results emphasize the involvement of mucosal MCs in Th1-mediated disorders.

## Background

The inarguable key role of mast cells (MCs) in allergic disorders is well established [1]. Moreover, there is a growing evidence for the important functions of MCs in host defense against parasitic and bacterial pathogens [2], autoimmune diseases [3], and other chronic inflammatory processes such as atherosclerosis [4]. Immature MC progenitors migrate from the bone marrow via the peripheral blood into the tissue, where they undergo final maturation. Consequently, human MCs can be developed *in vitro* from bone marrow, cord blood, and peripheral blood cells in the presence of Stem cell factor (SCF), the essential MC growth factor required for MC development and maintenance [1]. However, the phenotype of mature tissue-resident MCs is dictated by the local microenvironment. Therefore, MCs of different tissues exhibit remarkable differences in biochemical and functional properties [1]. The functional properties of MCs are significantly altered under pathological conditions and the cytokine milieu is considered a key factor in this regulation. Th2 type cytokines such IL-3, IL-4, IL-5, and IL-9 have been shown to enhance human MC growth, to increase degranulation and to enhance the production of eicosanoids and many cytokines upon FcϵRI-crosslinking [1,5-7]. These findings mainly explain elevated MC numbers and enhanced activity in Th2 type disorders [1,8].

INF-γ is the hallmark cytokine of Th1-mediated disorders such as auto-immune diseases and Crohn’s disease. It is mainly produced by NK and T cells and acts on many cells including dendritic cells, macrophages, and MCs [9]. For a long time INF-γ has been mainly considered as an inhibitory factor for MCs. Reports on rodent MCs have shown that INF-γ decreases MC development [10,11] and FcϵRI-dependent activation [12,13]. However, more recent studies using human peripheral blood-derived MCs indicate that INF-γ does not affect IgE-dependent degranulation and induces the expression of the FcγRI rendering MCs responsive to IgG-crosslinking [14]. There is conflicting data concerning the role of IFN-γ as a MC growth factor, since IFN-γ has been reported to either inhibit [15,16], promote [17], or to have no effect [15] on MC survival. These contradictory results might be explained by varying maturation states of the *in vitro* derived MCs. Kulka and Metcalfe showed that MC growth is strongly inhibited if INF-γ is added to early MC progenitors by both inhibiting proliferation and inducing apoptosis whereas the growth of differentiated peripheral blood-derived MCs was only slightly affected by INF-γ [15].

Similar findings have been described for other cytokines which is best exemplified by the effect of IL-4 on MCs. It is well established that IL-4 decreases the growth of early MC progenitors [7,15,18]. This effect seems to be turned to the opposite in more differentiated MCs. For late stage *in vitro* differentiated MCs it has been reported that IL-4 slightly decreases [7], slightly increases [16], or strongly increases [15] MC growth. Importantly, besides stem cell factor (SCF), IL-4 is the most potent cytokine inducing MC proliferation and enhancing IgE-dependent mediator release in tissue-derived human MCs [7,19].

These findings reveal the complex biology of human MCs, which can only be understood in detail if the results of studies on human MCs derived from different sources are compiled. Here we studied the regulatory effects of IFN-γ on purified human mucosal MCs. We demonstrate a dual role of INF-γ in the regulation of MC growth. Addition of IFN-γ inhibits the proliferation of MCs cultured under optimal growth conditions on the one hand. On the other hand, in the absence of the essential MC growth factor SCF, IFN-γ partly rescues MC survival by inhibiting apoptosis. Moreover, we demonstrate profound effects of IFN-γ on the functional properties of MCs. While it does not alter the expression or responsiveness of the high affinity FcϵRI, IFN-γ upregulates expression levels of FcγRI on MCs. Crosslinking of surface-bound monomeric IgG on IFN-γ treated MCs induced degranulation and release of histamine as well as production of leukotriene (LT)C_4,_ whereas IFN-γ naïve MCs failed to degranulate and released only low levels of (LT)C_4_. Thus, IFN-γ represents an important regulator of mature human MCs, which may have important implications during Th1 mediated diseases.

## Results

### IFN-γ has a dual role for MC growth by differentially regulating MC proliferation and apoptosis

**Figure 1:**
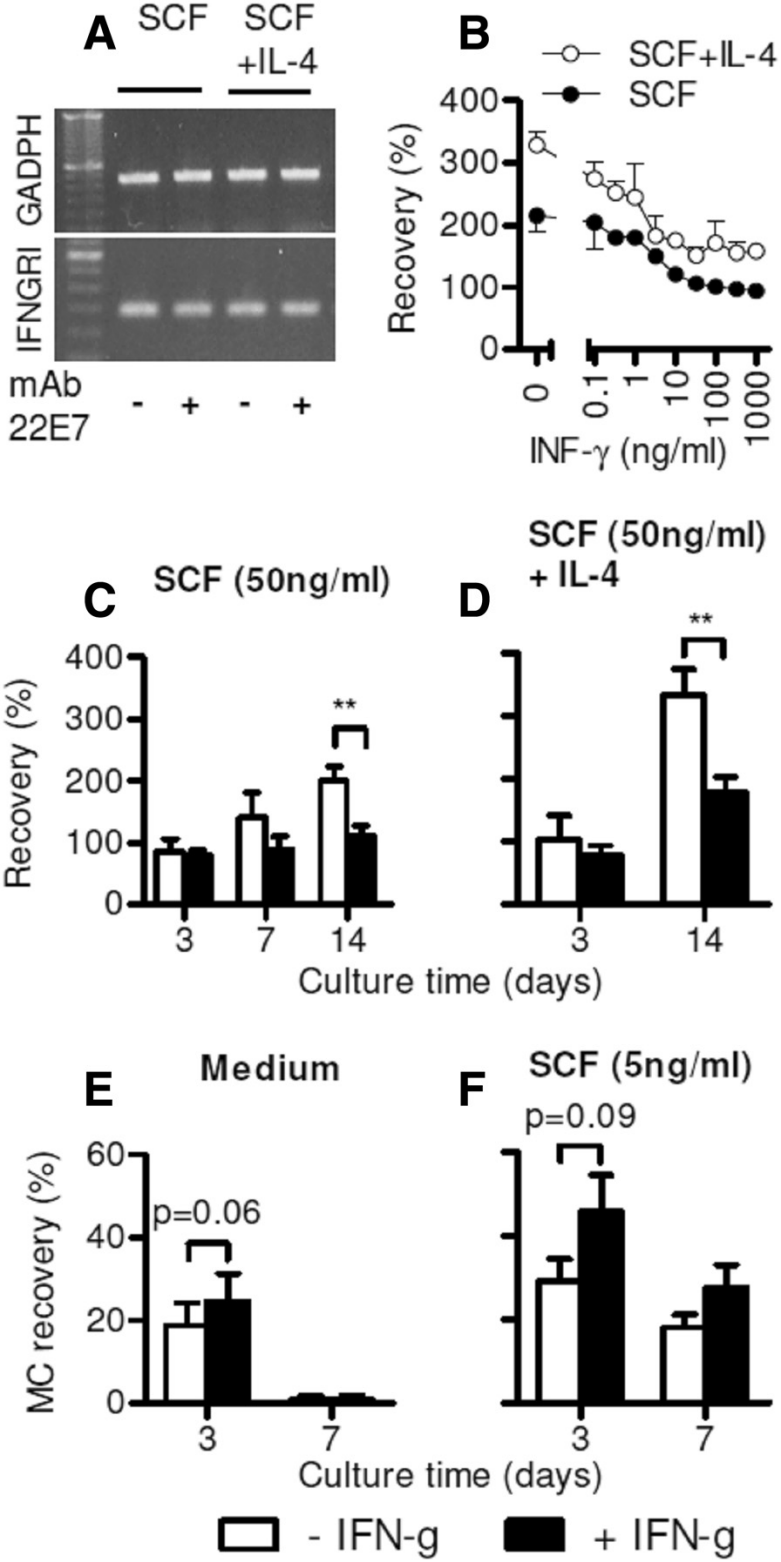
**Effects of IFN-γ on mast cell growth in vitro. (A)** mRNAs encoding for GAPDH and IFNGRI detected by RT-PCR in MCs following culture for 14 days in the presence of SCF (50 ng/ml) or SCF and IL-4 (2 ng/ml) and subsequent challenge for 60 minutes with mAb22E7 (+) or buffer control (−). **(B)** Dose-dependent growth inhibition of MCs by IFN-γ. MCs were cultured for 14 days in the presence of SCF (50 ng/ml) or SCF and IL-4 (2 ng/ml) and without or with different concentrations of IFN-γ (indicated in the graph). One representative experiment performed in duplicates out of two is shown. **(C-D)** MC recovery after culture in different conditions for indicated time periods with or without the addition of IFN-γ (100 ng/ml). **(C)** Culture in medium alone ± IFN-γ (n = 5). **(D)** Culture in the presence of SCF (5 ng/ml) ± IFN-γ (n = 5). **(E)** Culture in the presence of SCF (50 ng/ml) ± IFN-γ (n = 7 for day 3 and day 14, n = 5 for day 7). **(F)** Culture in the presence of SCF (50 ng/ml) + IL-4 (2 ng/ml) ± IFN-γ (n = 7). All data are shown as mean ± SEM. **p < 0.01.

IFNGRI mRNA is expressed in human intestinal MCs and the expression level is not changed by IL-4 or by FcϵRI-crosslinking (Figure 1A). We tested whether IFN-γ has an impact on MC growth. To this end, we cultured MCs in the presence of optimal concentrations of SCF or SCF and IL-4 and various concentrations of IFN-γ. IFN-γ decreased the MC recovery after 14 days in a dose-dependent manner with a maximal effect at concentrations ≥ 30 ng/ml (Figure 1B). To analyze the time kinetic of this effect we determined MC numbers at day 3, 7, and 14. The growth inhibitory effect of IFN-γ, which was significant at day 14, could not be detected at day 3 and was not significant at day 7, although there was already a strong tendency towards reduced MC numbers in cultures with IFN-γ (Figure 1C/D). To better understand the role of IFN-γ during MC growth, we lowered the concentrations of the dominant growth factor SCF. Intestinal MCs cultured in medium alone or in the presence of suboptimal concentrations of SCF (5 ng/ml), died completely or were strongly reduced in numbers, respectively. Surprisingly, IFN-γ partly inhibited the decline of MC numbers under these culture conditions, although the effect did not reach statistical significance (Figure 1E/F).

**Figure 2:**
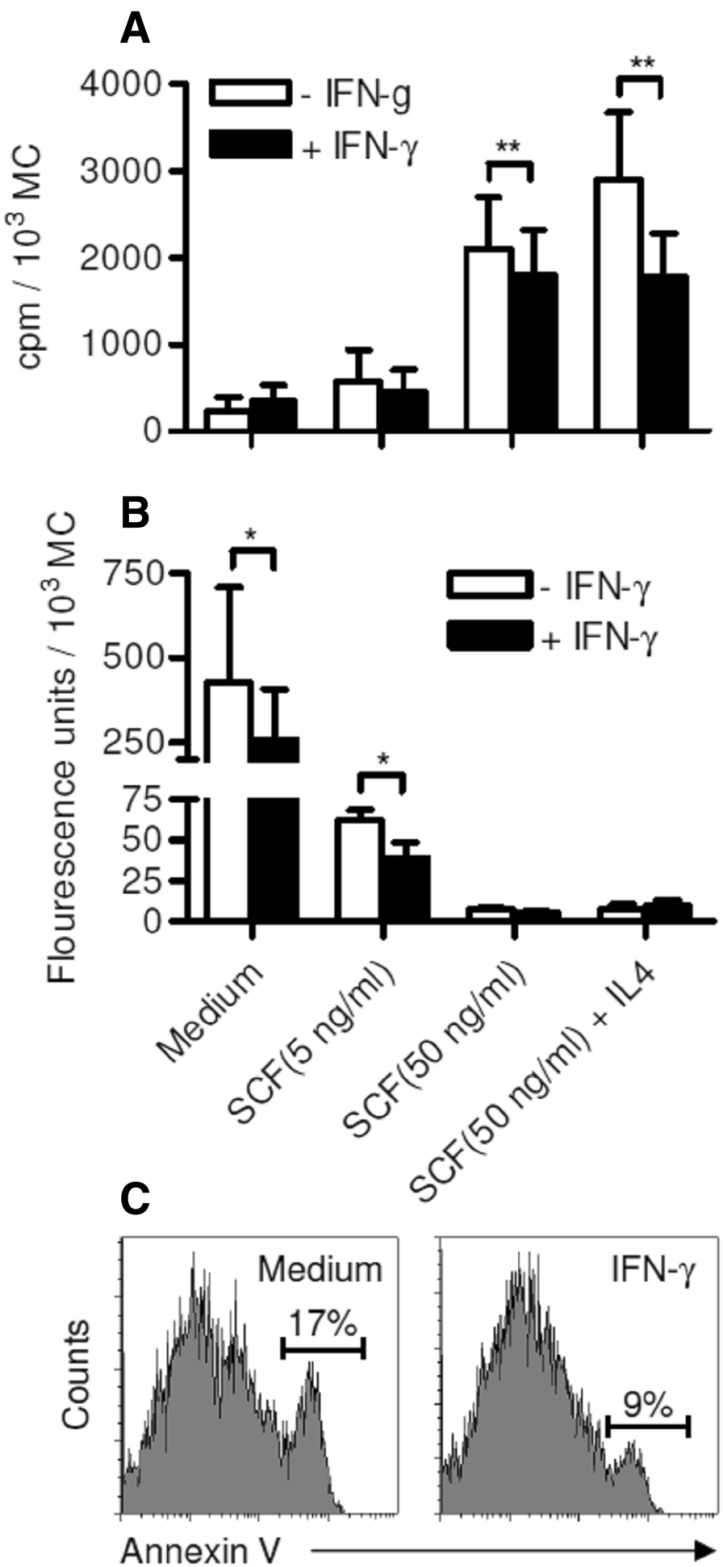
**Effects of IFN-γ on proliferation and apoptosis of intestinal MCs. (A/B)** MCs were cultured for 3 days in the conditions described in Figure 1C-F. **(A)** [^3^H] thymidine (0.5 Ci/well) was added for the final 12 h of culture to assess MC proliferation. Incorporation of [^3^H]thymidine into the cells was measured and is depicted as cpm/10^3^ MCs. Mean ± SEM of 5 (medium and SCF 5 ng/ml) or 7 independent experiments (SCF 50 ng/ml and SCF + IL-4) performed in duplicates. **(B)** Caspase 3/7 activity was determined and is shown as fluorescence units/10^3^ MCs. Mean ± SEM of 5 independent experiments performed in duplicates. **(C)** Surface binding of annexin V measured by FAVS following culture of MC for 3 days with IFN-γ (100 ng/ml) or medium alone. One representative experiment out of two is shown. *p < 0.05; **p < 0.01.

To further investigate the underlying mechanisms of the pro-survival effect of IFN-γ we studied ^3^H-Tymidin incorporation, as a measure of MC proliferation as well as Caspase 3/7 activity to determine apoptosis. MCs cultured in medium alone or in the presence of 5 ng/ml SCF generally showed very low rates of ^3^H-Tymidin incorporation, which was unchanged by IFN-γ treatment. Confirming previous results, we found high rates of ^3^H-Tymidin incorporation in MCs cultured with 50 ng/ml SCF, which was even more pronounced in MCs cultured in the presence of 50 ng/ml SCF and 2 ng/ml IL-4 [7,20]. MC proliferation under these conditions was significantly inhibited if IFN-γ was added to the culture medium. This effect was observed after 3 days (Figure 2A) and 7 days of culture (not shown), although the decrease in cell numbers was significant only after 14 days (Figure 1C/D). This might be explained by the overall slow proliferating phenotype of MCs *in vitro*, which makes changes in the proliferation levels only detectable by cell counting at later time points [7].Caspase 3/7 activity was low in MCs cultured in the presence of 50 ng/ml SCF with or without IL-4. IFN-γ did not influence caspase 3/7 activity under these conditions. In contrast, IFN-γ significantly inhibited caspase 3/7 activity in MCs cultured in the presence of 5 ng/ml or without SCF (Figure 2B). The anti-apoptotic effect of IFN-γ in MCs was further confirmed by FACS analysis of annexin V binding, a marker for early apoptosis (Figure 2C).

### IFN-γ does not change histamine content, FcϵRI expression, and FcϵRI-dependent mediator release

**Figure 3:**
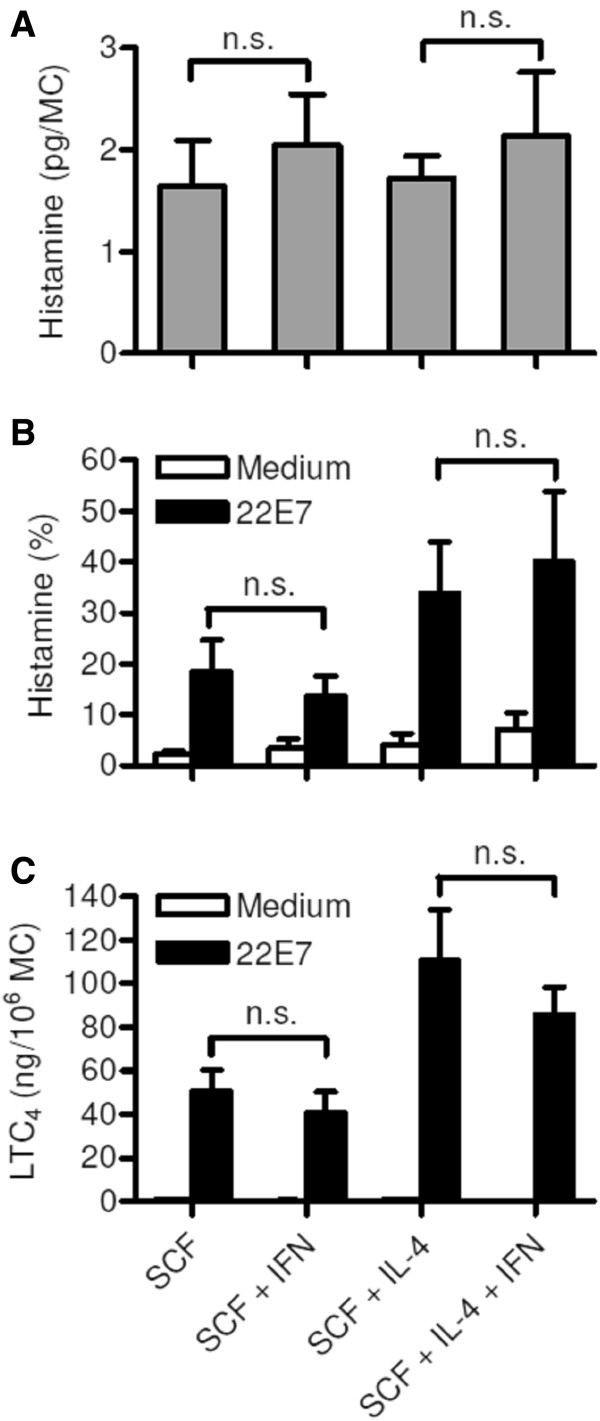
**IFN-γ does not change histamine content and FcϵRI-dependent mediator release of MCs.** MCs were cultured for 14 days in medium supplemented with SCF (50 ng/ml), with SCF and IFN-γ (100 ng/ml), with SCF and IL-4 (2 ng/ml), or with all three cytokines. **(A)** Total cellular histamine content of MC measured after cell lysis (n = 7). **(B)** Histamine (n = 5) and **(C)** LTC_4_ (n = 4) release after stimulation of MCs by FcϵRI crosslinking for 30 min using mAb 22E7 (100 ng/ml). All data are shown as mean ± SEM; n.s. = non significant.

**Figure 4:**
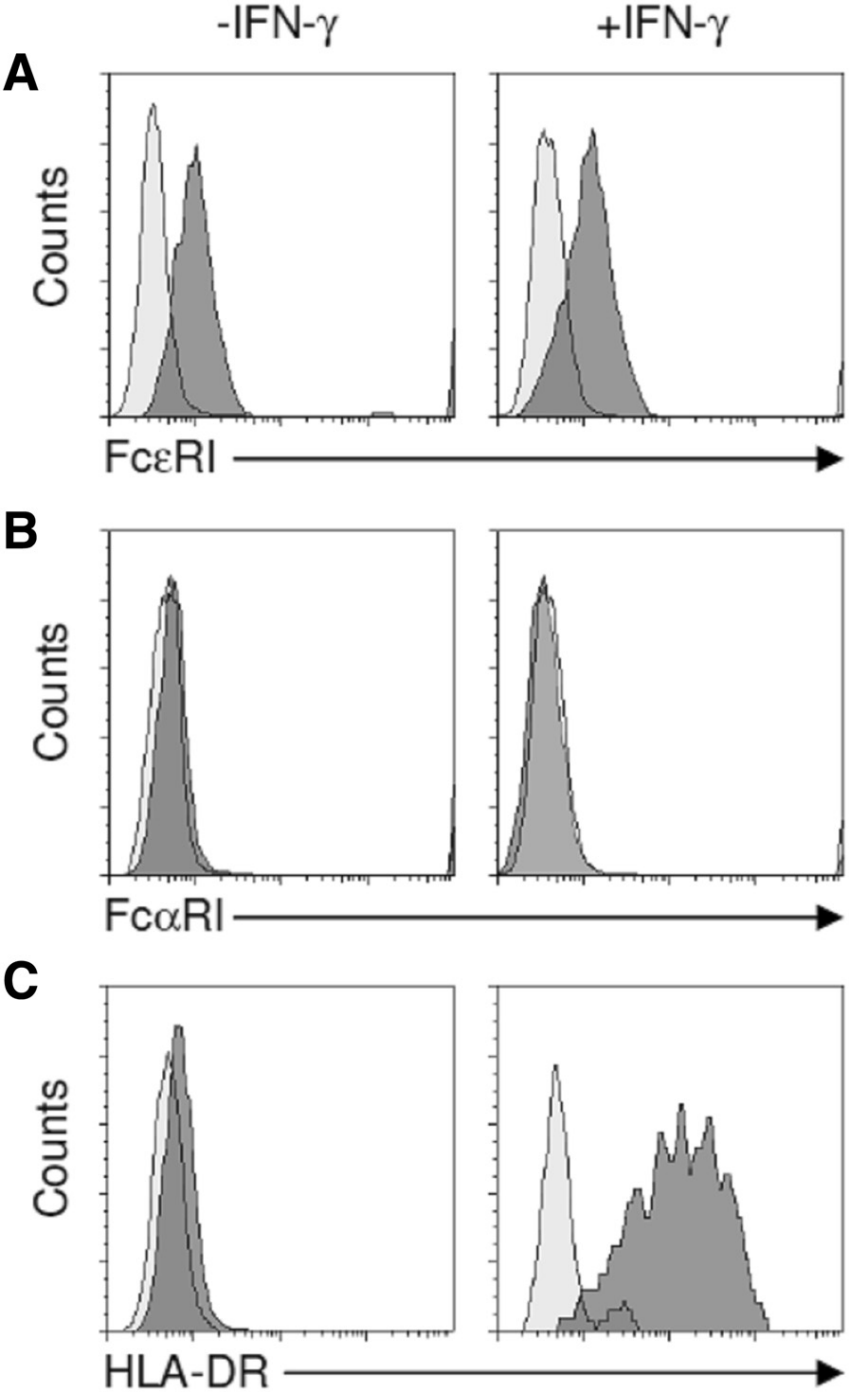
**Expression of FcϵRI, FcαRI, and HLA-DR in human intestinal mast cells cultured with or without IFN-γ.** MCs were cultured for 3 weeks in the presence of SCF (50 ng/ml) with or without addition of IFN-γ (100 ng/ml) to the culture medium for the last 3 days. Surface expression of **(A)** FcϵRI, **(B)** FcαRI, and **(C)** HLA-DR was analyzed by FACS. One experiment out of three is shown.

We reported previously that cytokines affecting MC growth such as IL-3, IL-4, and TGF-β also regulate the functional response of intestinal MCs to FcϵRI-crosslinking [5,21,22]. Here we show that IFN-γ has no effect on the histamine content in MCs (Figure 3A), and the release of histamine and LTC_4_ upon FcϵRI-crosslinking (Figure 3B/D). Our results confirms that IL-4 upregulates FcϵRI-dependent histamine and LTC_4_ liberation [7] and this effect is not counter-regulated by IFN-γ (Figure 3B/D). In line with the functional data, we found that IFN-γ did not change the expression level of the FcϵRI (Figure 4A). FcαRI was not detectable in intestinal MCs cultured with SCF alone or SCF + IFN-γ (Figure 4B). IFN-γ strongly induced the expression of the MCH class II protein HLA-DR in human intestinal MCs (Figure 4C) confirming studies on rodent MCs, the human MC line HMC-1 and human progenitor cell-derived MCs [23-26].

### IFN-γ upregulates FcγRI expression and the functional response to IgG-crosslinking

**Figure 5:**
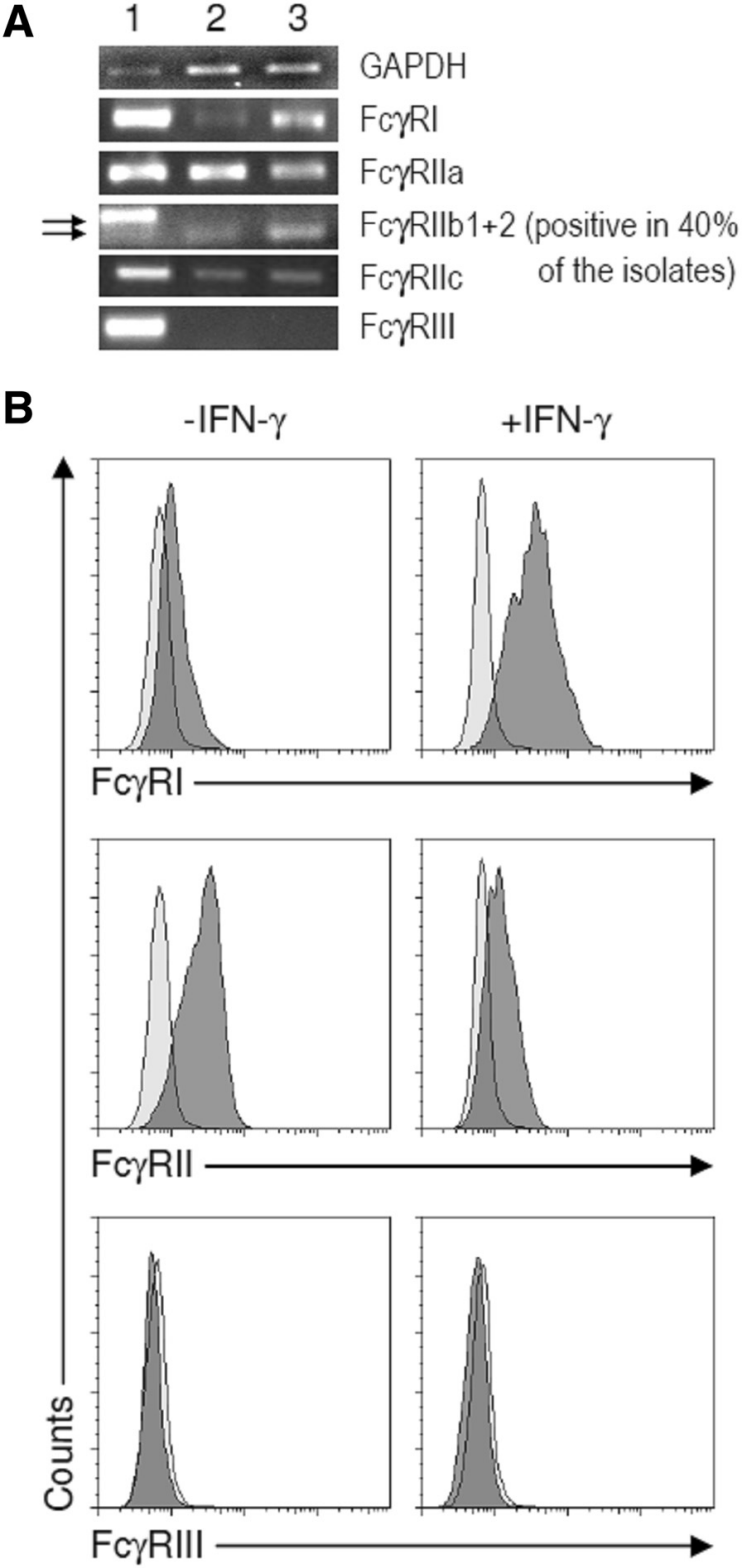
**Expression of Fcγ receptors in human intestinal mast cells. (A)** PCR detecting mRNAs encoding for GAPDH, FcγRI, FcγRIIa, FcγRIIb, FcγRIIc, and FcγRIII. For FcγRIIb the two isoforms FcγRIIb1 (343 bp) and FcγRIIb2 (289 bp) were detected. Lane 1: PBMCs (positive control). Lane 2: intestinal MCs cultured in the presence of SCF (50 ng/ml). Lane 3: intestinal MCs from the same donors cultured in the presence of SCF and IFN-γ (100 ng/ml) for the last 3 days. One experiment out of 10 is shown. FcγRIIb expression was found in MCs derived from 4 out of 10 donors (data not shown). **(B)** Surface expression of FcγRI (CD64), FcγRII (CD32), and FcγRIII (CD16) following culture of MC in the presence of SCF with or without the addition of IFN-γ (100 ng/ml) for the last 3 days. One experiment out of three is shown.

**Figure 6:**
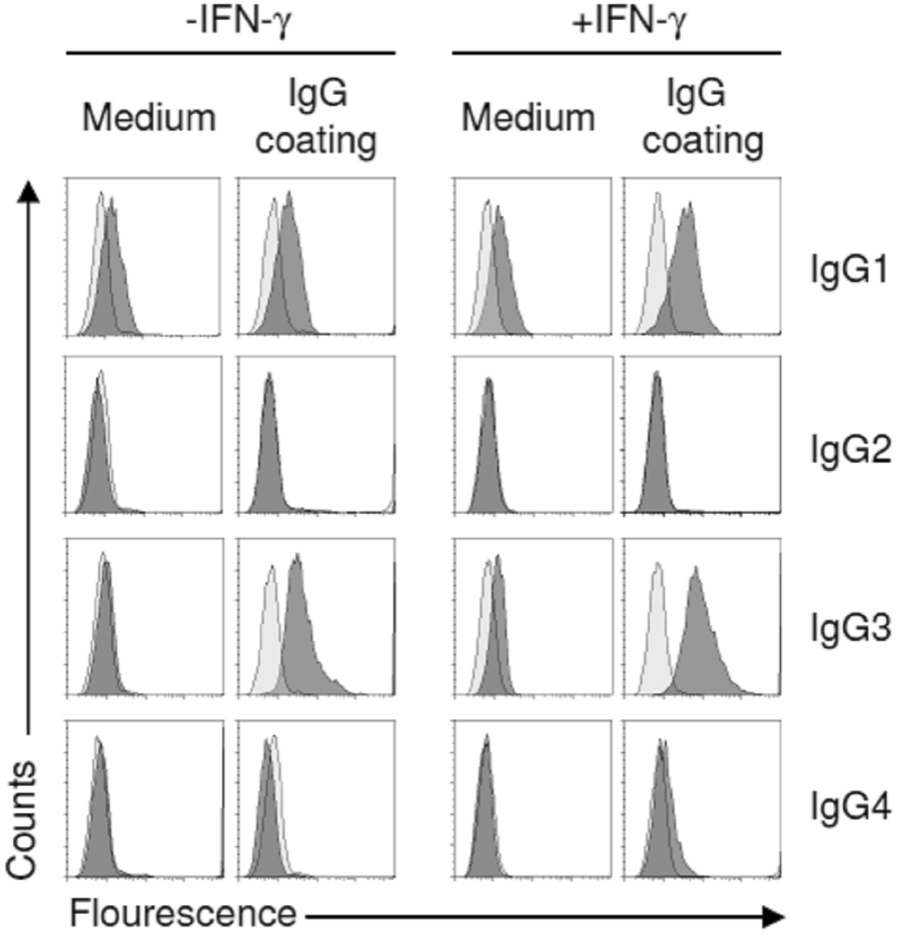
**Binding of IgG subtypes on intestinal MCs.** MCs were cultured in the presence of SCF with or without addition of IFN-γ (100 ng/ml) for the last 3 days. Then, MCs were incubated with IgG1-4 (500 μg/ml), respectively, or buffer control for 60 min. Binding of IgGs was analyzed by FACS with mAbs specific for the respective IgG subtype (dark gray peak). Isotype control (bright gray peak). One experiment out of three is shown.

We sought to investigate Fcγ receptor expression and function and the regulatory impact of IFN-γ in human intestinal MCs. We found that human intestinal MCs express mRNA for FcγRI, FcγRIIa, and FcγRIIc at steady state (Figure 5A). Very low amounts of FcγRIIb1 and higher levels of FcγRIIb2 expression was only detected in 4 out of 10 MC preparations derived from different donors (Figure 5A and data not shown) and FcγRIII expression was not detectable in any of the samples (Figure 5A). FACS analysis confirmed the mRNA results as we detected FcγRI and FcγRII (subtypes could not be differentiated by the mAb), but no FcγRIII expression on the cell surface. IFN-γ increased the expression of the FcγRI on mRNA and protein level (Figure 5A/B). FcγRII expression of intestinal MCs was downregulated by IFN-γ (Figure 5B). The mRNA analysis suggests that this regulation was dependent on the decreased expression of FcγRIIa, whereas FcγRIIc expression was not affected (Figure 5A). IL-4, IL-10, G-CSF, or GM-CSF had no influence on the expression level of FcγRs in human intestinal MCs (data not shown).Next, we investigated the binding of IgG subtypes on intestinal MCs by flow cytometry. We found that MCs bound myeloma IgG1 and IgG3 which was significantly increased by IFN-γ. Marginal binding of IgG4 was found on IFN-γ treated MCs, whereas IgG2 binding was absent in all tested conditions (Figure 6).

**Figure 7:**
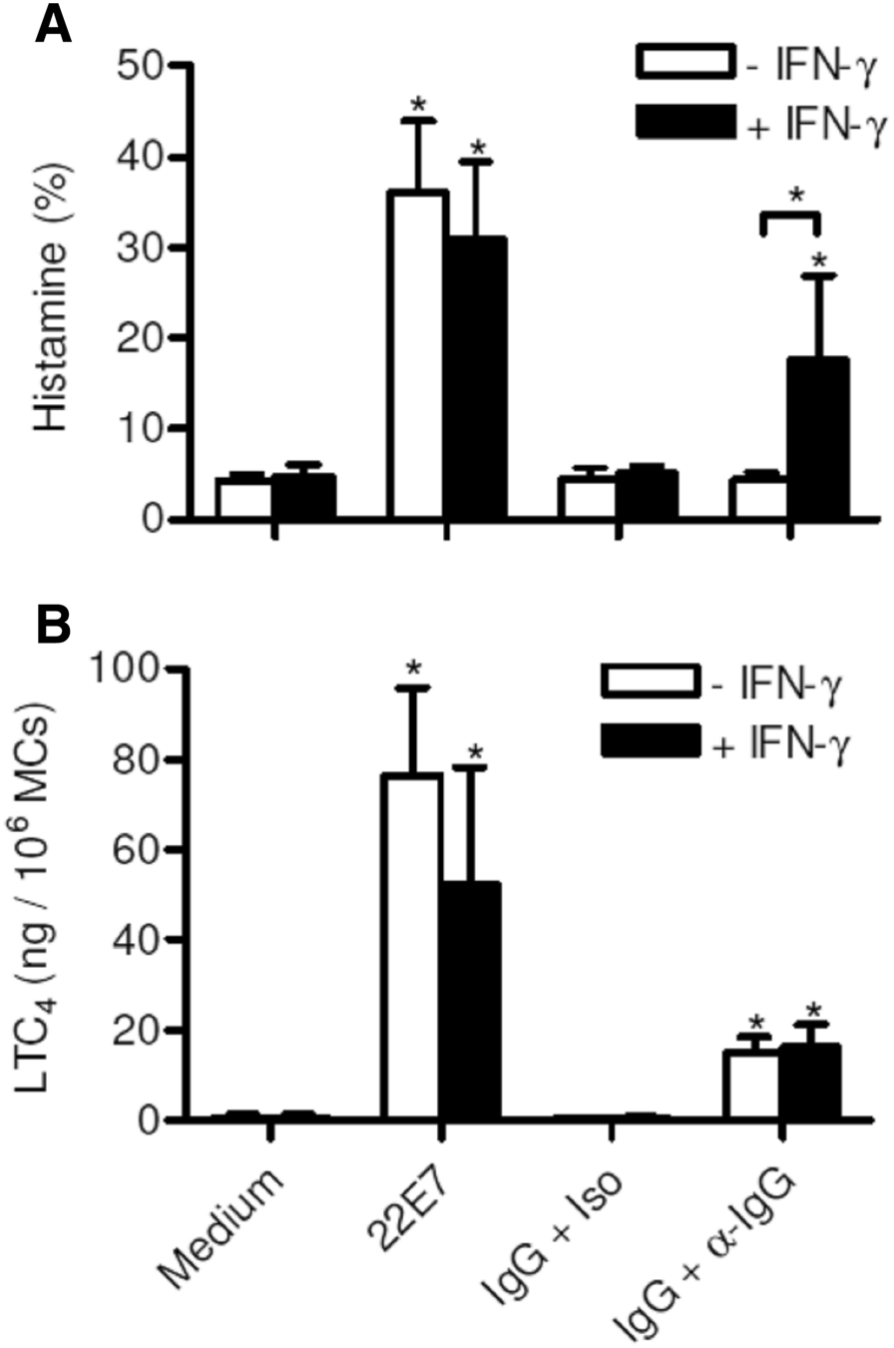
**Histamine and LTC**_**4 **_**release from intestinal MCs after IgG-crosslinking.** MCs were cultured with SCF and with or without IFN-γ (100 ng/ml) for 2 days. The last 24 h plasma IgG (10 μg/ml) was added to the culture medium if indicated. Then, MCs were washed and challenged with an anti-IgG mAb or and isotype control mAb for 60 min. FcϵRI-crosslinking induced by mAb 22E7 served as positive control and non-treated MCs as negative control. **(A)** Histamine (n = 6) and **(B)** LTC_4_ (n = 4) were measured in the supernatants. Means ± SEM are shown. *p < 0.05 (in comparison to medium control if not indicated otherwise).

To characterize the functional response of human intestinal MCs to IgG-crosslinking we sensitized MCs with plasma IgG for 24 h and subsequently challenged the cells with an anti-human IgG mAb. Primary human MCs cultured in the absence of IFN-γ failed to release stored histamine upon FcγR activation. Interestingly, FcγR stimulation of IFN-γ treated MCs induced degranulation and rapid release of histamine contents (Figure 7A). IFN-γ-treated and untreated MCs produced similar amounts of LTC_4_ upon IgG-crosslinking (Figure 7B). In summary, these results indicate that IFN-γ induces upregulation of FcγRI expression and renders intestinal MCs responsive to IgG-induced degranulation, whereas IgG-dependent production of de-novo synthesized eiconsanoids is independent of IFN-γ.

## Discussion

In the present study, we identify IFN-γ as an important regulator of human intestinal MCs. IFN-γ decreases the proliferation of human intestinal MCs cultured in the presence of high doses of SCF and, even more importantly, in the presence of SCF + IL-4. We further found that IFN-γ does not have an impact on the apoptosis rates of human intestinal MCs cultured in the presence of 50 ng/ml of SCF with or without IL-4. In contrast, we found that IFN-γ reduces apoptosis in MCs cultured in the presence of suboptimal concentration of SCF or in medium alone. These findings explain some of the discrepancies observed in previous studies. IFN-γ inhibits the development of MCs in vitro by inhibiting proliferation and inducing apoptosis of MC progenitors [11,15,27]. However, the effects of IFN-γ on differentiated MCs seemed to be contradictory in different studies. IFN-γ has been shown to act on human cord-blood-derived MCs in one study by decreasing survival and proliferation [16] and in another study by promoting survival and inhibiting apoptosis [17]. The main difference in the two study protocols is the presence of 100 ng/ml SCF in the MC cultures in the first report in contrast to the complete lack of growth factors during IFN-γ exposure in the latter publication. Therefore, our data confirm the results of both studies and reveal the divergent effects of IFN-γ on MC survival depending on the cross-talk with other growth factors. A recent study suggested that IFN-γ does not affect the growth of human peripheral blood-derived MCs. In this study MCs were differentiated for 8 weeks in the presence of SCF and then further cultured with SCF and SCF + IFN-γ for additional 7 days. Proliferation was tested by CSFE-dilution within the last 7 days of the culture [15]. We found that intestinal MCs cultured in the presence of SCF only proliferate slowly. Consequently, the effect of IFN-γ on proliferation was readily detectable by measuring [^3^H] thymidine incorporation, whereas significant differences in MC numbers were found only after 14 days of culture. Also in the study performed by Kulka and Metcalfe the differentiated MCs exhibited a low proliferation rate and in the presence of SCF only 28.5% of the cells divided within the 7 day of the investigation period. In the presence of SCF + IFN-γ the division rate dropped to 19.3% [15]. Inhibition of proliferation of around 30% correlates very well with the findings in our study.

The *in vivo* effect of IFN-γ on MCs is supposedly influenced by the microenvironment such as the local concentrations of other growth factors such as SCF or IL-4. These factors vary in physiological and different pathophysiological conditions. One might expect that IFN-γ counter-regulate massive MC proliferation in Th2-type inflammatory diseases or dysregulated c-kit (SCF receptor) activation as it has been found in mastocytosis. Indeed, IFN-γ treatment has been shown to improve the clinical symptoms of a mastocytosis patient [28]. On the other hand, increased IFN-γ levels such as found in autoimmune diseases may stabilize or even elevate MC numbers in a less proliferation permissive environment. This may explain the high MC numbers frequently found in typical Th1 disorders such as rheumatoid arthritis [29] or psoriasis [30].

Recent studies employing mouse models have emphasized the role of FcγR-dependent activation of MCs in the development of immune-complex associated autoimmune diseases [3,31]. However, mouse MCs express only the low-affinity IgG receptors FcγRII and FcγRIII, but not the high-affinity FcγRI. We show here that human intestinal MCs cultured in the presence of SCF alone express low levels of FcγRI. IFN-γ treatment strongly increased its expression. Our findings are in agreement with recent immuno-histological studies demonstrating that in a small subset of intestinal MCs FcγRI is expressed during homeostasis, but that the frequency of FcγRI^+^ MCs is strongly increased in patients with Crohn’s disease, a disease which is associated with increased IFN-γ levels [32]. The FcγRI^+^ expression profile in intestinal MCs is very similar to what has been reported for MCs derived from peripheral and cord blood as well as skin and synovial MCs, which express no or very low levels of FcγRI^+^ after culture with SCF [14,33-35]. However, in peripheral blood-dervied MCs FcγRI expression can be induced by IFN-γ [14], and immuno-histological studies showed that skin MCs are negative for FcγRI expression during homeostasis, while FcγRI expression can be detected in MCs of tissue samples derived from patients with psoriasis [36]. After sensitization with IgG, we detected the binding of IgG1 and IgG3 on the surface of MCs, which was increased after IFN-γ exposure. Monomeric IgG only bind to the high affinity FcγRI with the highest affinities for IgG1 and IgG3, whereas low affinity FcγRII and III preferentially bind IgG complexes [37]. Enhanced binding of IgG1 and IgG3 to MCs cultured in the presence of IFN-γ is, therefore, very likely caused by the increased expression of FcγRI. We found that IgG-crosslinking triggers histamine release only in IFN-γ-treated MCs, but not in MCs cultured without IFN-γ. This confirms recent studies showing that peripheral blood-derived MCs cultured in the presence of IFN-γ release histamine and de-novo produced eicosanoids such as LTC_4_ and PGD_2_ and several cytokines such as IL-3, IL-5, IL-6, IL IL-13, TNF, and GM-CSF after IgG-crosslinking [14,38]. In contrast to peripheral blood-derived MCs, intestinal MCs released LTC_4_ upon IgG-crosslinking without IFN-γ pre-culture. The amount of LTC_4_ release was not increased in IFN-γ-treated MCs. This suggests that LTC_4_ production can be induced by low level FcγRI aggregation, whereas the threshold for the induction of MC degranulation is higher and requires more pronounced FcγRI-dependent signals.

Furthermore, we found that intestinal MCs express mRNA for FcγRIIa and FcγRIIc, but not FcγRIII. FcγRIIb was expressed in 40% of the MC isolates. The expression of FcγRIIa mRNA was downregulated by IFN-γ, which correlated with increased detection of FcγRII surface expression using a Pan-FcγRII mAb. The expression profile of low-affinity FcγRs differs in MCs derived from different sources. Peripheral-blood derived MCs express FcγRIIa, FcγRIIb, and FcγRIII, but no FcγRIIc [39]. Cord-blood derived MCs encode mRNA for FcγRIIa, FcγRIIb, and FcγRIIc, but no FcγRIII; however, only FcγRIIb protein expression could be detected [34]. In contrast, skin MCs express only FcγRIIa and no FcγRIIb, FcγRIIc, and FcγRIII [33]. Synovial MCs express FcγRIIa, FcγRIIb, and FcγRIIc, and no FcγRIII, however; they dowregulate FcγRIIb expression upon culture [35]. The low-affinity IgG receptors FcγRIIa, FcγRIIc, FcγRIII are activating receptors that signal as FcγRI via immunoreceptor tyrosin-based activation motifs (ITAM) and are aggregated by immune-complexes. They are expressed on many innate immune cells and exert various effector pathways, such as phagocytosis, antibody-dependent cellular cytotoxicity, and the release of chemotactic and pro-inflammatory mediators [40]. Human skin and synovial MCs, however not peripheral-blood derived MCs, have been shown to degranulate and secrete eicosanoids and cytokines upon aggregation of FcγRIIa [33,35,39]. However, recently FcγRIIa has been suggested to decrease IgE-mediated activation of basophils by a yet undefined mechanism [41]. FcγRIIb is an inhibitory Fc receptor and transmits its signals via immunoreceptor tyrosin-based inhibitory motifs (ITIM) [42]. FcγRIIb inhibits IgE-induced activation of human basophils and cord blood-derived MC [34,43]. Polymorphisms in the promoter and transmembrane region causes decreased expression of FcγRIIb and are linked to lupus erythematosus. We found inconsistent expression of FcγRIIb in human intestinal MCs which may be explained by genetic variability of the donors [44]. The cellular response after aggregation of low-affinity IgG receptors on intestinal MCs has not been addressed in the current study and requires further investigation.

## Conclusion

In summary, we define IFN-γ as an important regulator of human intestinal MCs. IFN-γ provides inhibitory signals such as blocking extensive MC proliferation and activating signals such as decreasing apoptosis in starving MCs, inducing the expression of FcγRI and MHC class II molecules, and rendering MCs more responsive to IgG-crosslinking. This differential regulation of MCs might be of particular importance for MC function during Th1-type mediated diseases [3,31].

## Methods

### Isolation, purification, and culture of human intestinal MCs

MCs were isolated from surgical tissue specimens (macroscopically normal tissue) derived from individuals who underwent bowel resection because of cancer. Written informed consent was obtained from all patients at least 24 h before surgery. The study was approved by the local ethical committee of the Medical School of Hannover, Germany, where the study was performed. The methods of mechanical and enzymatic tissue dispersion yielding single cell preparations have been described [45]. After overnight culture of the cell suspension in culture medium (RPMI 1640 supplemented with 10% heat-inactivated fetal calf serum, 25 mM HEPES, 2 mM glutamine, 100 μg/ml streptomycin, 100 μg/ml gentamycin, 100 U/ml penicillin, and 0.5 μg/ml amphotericin; all from Invitrogen, Karlsruhe, Germany) *c-kit* expressing MCs were enriched by positive selection using magnetic cell separation (MACS™ system, Miltenyi Biotech, Bergisch-Gladbach, Germany) and the anti-*c-kit* mAb YB5.B8 (5 ng/ml, PharMingen, Hamburg, Germany) as described [45]. The fraction containing the *c-kit*-positive cells (MC purity 50–90%) was cultured at a density of 1 – 2 × 10^5^ MC/ml in the presence of SCF (50 ng/ml, Amgen, Thousand Oaks, CA) for two weeks to obtain >98% pure MCs. MCs were harvested, washed, and further cultured for 3–14 days in 96-well plates (2 × 10^4^/well) without cytokines or in the presence of SCF, IL-4 (Novartis, Vienna, Austria) and/or IFN-γ (Imukin, Boeringer Ingelheim Pharma GmbH & Co., Ingelheim, Germany) at indicated concentrations. Once a week half of the culture medium was exchanged and cytokines were supplemented again.

### Detection of apoptosis and proliferation

Apoptotic MCs were visualized by FACS using APC-conjugated annexin V (Becton Dickinson) Caspase 3/7 activity was measured with the Apo-ONE™ Homogeneous Caspase 3/7 Assay (Promega, Madison, WI). For the analysis of cell proliferation MCs were cultured in 96-well plates (2 × 10^4^ per condition) in the presence of indicted cytokines for 3 or 7 days. The cultures were pulsed with 1 μCi of [^3^H] thymidine (Amersham International, Amersham, UK) per well for the final 12 h. The cells were harvested on unifilter plates by using an automatic cell harvester (FiltermateTM196). [^3^H] thymidine corporation was measured as counts per min on a Beckman Topcount.

### RNA preparation and RT-PCR

Total RNA was prepared from 5–10 × 10^4^ MCs and RT-PCR was performed as described [22]. The following primers were used for RT-PCR: glyceraldehyde 3-phosphate dehydrogenase (GAPDH; 5′-CAT CAC CAT CTT CCA GGA GC-3′; 5′-GAG GCA GGG ATG ATG TTC TG-3); IFNGRI (5′-CCA TCT CGG CAT ACA GCA AA-3′; 5′-CTC AGT GCC TAC ACC AAC TA-3′); FcγRI (5′-CTT CTA CAT GGG CAG CAA GA-3′; 5′-GTT CTC TGG GTG ACA ATA CG-3′); FcγRIIa (5′-CAG CAT GGG CAG CTC TTC-3′; 5′-CAC ATG GCA TAA CGT TAC-3′); FcγRIIb1/2 (5′-ATT GTT GCT GCT GTA GTG GCC-3′; 5′-GAA ACC TTC TCT TTT GGA ACT-3′); FcγRIIc (5′-TCT AGA TGA CCA CAT GGC ATA ACG-3′; 5′-CCT GGA CGT CAA ATG ATT GCC ATC-3′); FcγRIII (5′-CTT CTG GGA TAA GTG GAC TC −3; 5′-CTT CAT GGT TAG TGG TTC GTC-3).

### Flow cytometry

FACS staining was performed as described recently [7]. MCs were labeled with primary antibodies anti-CD16-PE (Becton Dickinson, San Jose, CA), anti-CD32-PE, anti-CD64-PE, anti-CD89 (Caltag Laboratories, Hamburg, Germany), FcϵRI α-chain (mAb 22E7, Hoffmann-La Roche, kindly provided by R. Chizzonite), anti-HLA-DR, (DAKO A/S, Glostrup, Denmark) or appropriate isotype controls. Cells were washed and if purified primary Ab were used incubated with FITC-conjugated goat anti-mouse IgG1 (Southern Biotechnology, Birminghman, AL). FACS analysis was performed using the FACSCalibur™ system (Becton Dickinson). Analysis were performed using the FlowJo software (Treestar, Inc. Ashland, OR).

### Analysis of IgG binding to MCs

MCs were incubated for 60 min at 37°C with purified human myeloma IgG1, IgG2, IgG3, or IgG4 (500 μg/ml in Hepes/BSA buffer; myeloma IgG purchased from Calbiochem, SanDiego, CA) or were left untreated. After washing anti-human IgG1 (cloneJDC-1), anti-human IgG2 (cloneG18–21), anti-human IgG3 (cloneG18–3), or anti-human IgG4 (cloneJDC-14) (all from Becton Dickinson) were applied for 30 min at 4°C. Samples were washed and incubated with an FITC-conjugated goat anti-mouse IgG1 mAb (Southern Biotechnology). Binding of IgG subtypes were verified by FACS.

### Mediator release assay

For FcϵRI crosslinking, 2 × 10^4^ MCs were stimulated using the mAb 22E7 (100 ng/ml, 30 min, 37°C) directed against the high-affinity FcϵRI α-chain. For IgG-crosslinking, MCs were cultured in RPMI for 24 h in the presence of 10 μg/ml human plasma IgG (Calbiochem; plasma IgG were centrifuged before at 20,000 × g for 40 min at 4°C to remove aggregated molecules). Samples were washed twice and 2 × 10^4^ MCs were resuspended in 400 μl HACM buffer (20 mM HEPES, 125 mM NaCl, 5 mM, KCl, 0.5 mM, 1 mM CaCl_2_, 1 mM MgCl_2_, 0,1% BSA). Surface bound IgG was crosslinked by an anti-human IgG mAb (1 μg/ml, 30 min, 37°C, Becton Dickinson). Histamine (RIA, Coulter-Immunotech, Hamburg, Germany) and leukotriene C_4_ (LTC_4_, ELISA, Biotrend, Cologne, Germany) were measured in the supernatants.

### Statistics

All data in text and figures are expressed as mean ± SEM. Statistical differences between groups were determined using two-tailed Student’s t test.

## Abbreviations

LTC_4_: Leukotriene C_4_; MC: Mast cell; SCF: Stem cell factor.

## Competing interests

All authors have no competing financial interests.

## Authors’ contributions

Conceived and designed the experiments: GS, MB, AL, SCB. Performed the experiments: GS, MB, SK, TG. Analyzed the data: GS, MB. Collected tissues samples and isolated cells: GS, MB, SK, TG, LES. Wrote the paper: GS, LES, AL, SCB. All authors read and approved the final manuscript.
